# Brain lateralization for perceiving direction of motion is reversed in Williams syndrome and related to BUD23

**DOI:** 10.1038/s41598-025-94742-3

**Published:** 2025-06-05

**Authors:** Debra L. Mills, Li Dai, Julie R. Korenberg

**Affiliations:** 1 School of Psychology and Sport Science, Bangor University, Bangor, UK; 2 Department of Pediatrics, University of Utah, Salt Lake City, UT USA; 3 Aliri Bioanalysis, Salt Lake City, UT USA; 4 Department of Neurology, University of Utah, Salt Lake City, UT USA

**Keywords:** Genetics, Neuroscience, Psychology

## Abstract

Lateralized nervous system function is phylogenetically old but fundamentally important for human brain function. Although altered in developmental and psychiatric disorders, we know little about its genetics. To understand the genetic origins of hemispheric specialization, we investigated laterality in a genetic disorder, Williams Syndrome (WS), caused by ~ 27 deleted genes on 7q11.2. Using a multidisciplinary approach combining individuals’ molecular genetic, electrophysiological, and behavioral data, we identify reversed lateralization, from right to left hemisphere for perceiving direction of motion in WS and show hemispheric strengths are inversely correlated. Moreover, we correlate decreased transcript levels of the deleted gene *BUD23*, with strength of the reversed lateralization and with decreased performance in mental rotation, another right hemisphere lateralized function. The results implicate dosed *BUD23,* an 18S ribosomal RNA methyltransferase, in human brain laterality, support an evolutionary origin and provide altered lateralization as a novel mechanism for impaired cognition in genetic and behavioral disorders.

**Supplementary Information:**

The online version contains supplementary material available at 10.1038/s41598-025-94742-3.

## Introduction

Lateralization of brain function is one of the most enigmatic and ancient phylogenetic processes beginning ~ 550 mya at the dawn of chordate evolution^1–4^. In humans, the two hemispheres are differentially specialized for language and visuo-spatial abilities, to the left and right hemispheres respectively^5–8^. Such distinctions are associated with increased proficiency in typical development^3,9,10^ and laterality is altered in many neurodevelopmental and neuropsychiatric disorders such as autism spectrum disorder (ASD), Schizophrenia and dyslexia^11–14^. Nonetheless, little is known about genetic regulation of lateralized brain functions or their roles in cognition and behavior. Moreover, the genetic and neural networks altered in such disorders are largely unknown despite extensive evidence for shared, although poorly understood genetic risk^2^. Therefore, although elusive, the genes for brain lateralization may in turn provide genetic and neural insight into the origins of these conditions^15^.

The most common approach used to identify human genes linked with lateralized brain functions has been genome wide associate studies (GWAS) that correlate DNA variants associated with lateralized functions such as language or auditory processing and handedness^16–18^. Recent GWAS studies using the UK Biobank and other large databanks have identified hundreds of genes associated with structural brain asymmetries such as cortical thickness, gray matter volume, and subcortical volumes^8,17,18^. This approach is not without methodological challenges as GWAS studies are designed to query the 3,054 Mb constituting the entire genome for DNA variants associated with brain phenotypes such as volumes and neural activity. For most of these, the genetic cause is unknown. Consequently, they require large numbers of participants and any given variant typically accounts for only a small portion of variance. The issues of varying replicability and provision of group-based DNA risk without individual quantitative gene expression, limit inferences of functional consequences.

In contrast to GWAS where the effect size of any given gene variant is small, the effect size of a gene (or gene combination) is relatively large in humans with small deletions or duplications, and can greatly focus the search where the phenotype is known to be present. Therefore, although establishing reliable hemisphere-specific measures of lateralized cognitive processes at the individual level is far from trivial, combining these with genomic, genetic, and behavioral studies in a known human genetic condition is a powerful tool for parsing complex human behaviors and identifying specific genes influencing them. This is essential for validating and applying to therapeutics, the otherwise hypothetical roles of cell and rodent biology in human behavior.

To explore a genetic basis for specific lateralized brain functions we studied individuals with Williams syndrome (WS). WS is caused by a consistent deletion of ~ 25–28 genes over 1.5–1.8 Mb within chromosome region 7q11.2, associated with a consistent phenome^19–22^. This includes distinctive facial and physical features such as short stature and congenital heart disease, together with a consistent pattern of intellectual disabilities and positive social behavioral with heightened approach. Important for our work is that WS is associated with atypical patterns of lateralization^23^ with relative strengths in language (left hemisphere lateralized in TC (typical controls) and deficits in visuo-spatial skills (right hemisphere lateralized in TC) including detection of motion direction and mental rotation^20^. The exciting promise of WS for unravelling the first genes that are quantitatively involved in these complex human behaviors derives from their large effect sizes, i.e., the phenotypes occur with high frequency in WS. WS therefore provides a unique opportunity to drill down to single or small clusters of gene(s) and the neural systems that are altered in complex human behaviors. Previous research identifying the role of specific genes in the WS cognitive phenotype has related cognitive phenotypes only to the presence or absence of genes deleted in individuals with rare, hemizygous, partial deletions^24–28^ and implicated *GTF2IRD1* in visuo-spatial construction and both *GTF2IRD1* and *GTF2I* in cognition. However, this approach does not allow understanding the mechanisms leading from gene copy differences through development to brain or behavior. The reason is not obvious but is central for the leap from gene variant risk to quantitative outcomes needed for therapeutics. The cases with atypical deletions are rare, the gene expression may not reflect the genes deleted as expression may be altered by position effects, and the copy number is binary, present or absent. This allows for group comparisons but not variations among individuals with or without a given gene. Further, among those with typical WS deletions, the frequency of gene DNA sequence variants on the non-deleted chromosome as well as the subject numbers with such variants, are low. Moreover, the variation of human cognitive phenotypes is too high to allow their use in parsing genetic contributions to most outcomes involving brain activity or cognitive tasks at the individual level. In contrast, we utilized quantitative gene-specific transcription as a continuous trait and correlated it with quantitative, hemisphere-specific brain activity measured by ERP’s (event related potentials). ERP’s are averages of electrical signals from the brain that are measured at the scalp, at the speed of perception and thought. Further, the paradigm (see Methods) was designed to measure ERPs independently in the right (RH) and left (LH) hemispheres in order to detect “lateralization”, that is the differential ability between the two hemispheres to process and respond to stimuli. Here, we examined the ability of the RH and LH to respond differently to “direction of motion”, that is, to show a difference in brain activity to stimuli presented in the central visual field, that appear to move away from versus toward a given hemisphere. This ability is lateralized to the RH throughout evolution and in humans.

Our previous findings showed that the organization of brain activity linked to relative strengths in face^23,29–31^ and language^23,32,33^ processing was less lateralized in WS than in typical controls. Here we present ERP (event-related-potential) data showing reversed lateralization, from RH dominant in controls to LH in WS, for perceiving direction of motion, a hallmark deficit in WS^34–36^. RH laterality for direction of motion has its origins in early vertebrate phylogeny related to distinguishing predators from prey^3,4^ and has been cited as the original impetus for the evolution of brain laterality. To elucidate genetic and neural mechanisms at the level of the individual, we tested whether the strength of atypical hemispheric specialization in WS as measured from the ERP task, was related to decreased expression of specific deleted genes. We used three quantitative approaches related to gene expression in small cohorts of WS: array-based transcription and DNA methylation in cases with full deletions and the former in two atypical overlapping deletion cases. Results were then tested statistically using quantitative RTPCR in a larger cohort with full deletions. We found that the strength of atypical hemispheric specialization in WS measured by high-density ERPs was related to their lower efficiency of a second RH lateralized WS deficit, mental rotation. And both RH lateralized deficits and the reversed laterality for direction of motion in WS were correlated with decreased transcript levels, resulting from the deletion of specific genes (see methods) principally, *BUD23*.

## Results: brain activity

**Fig. 1:**
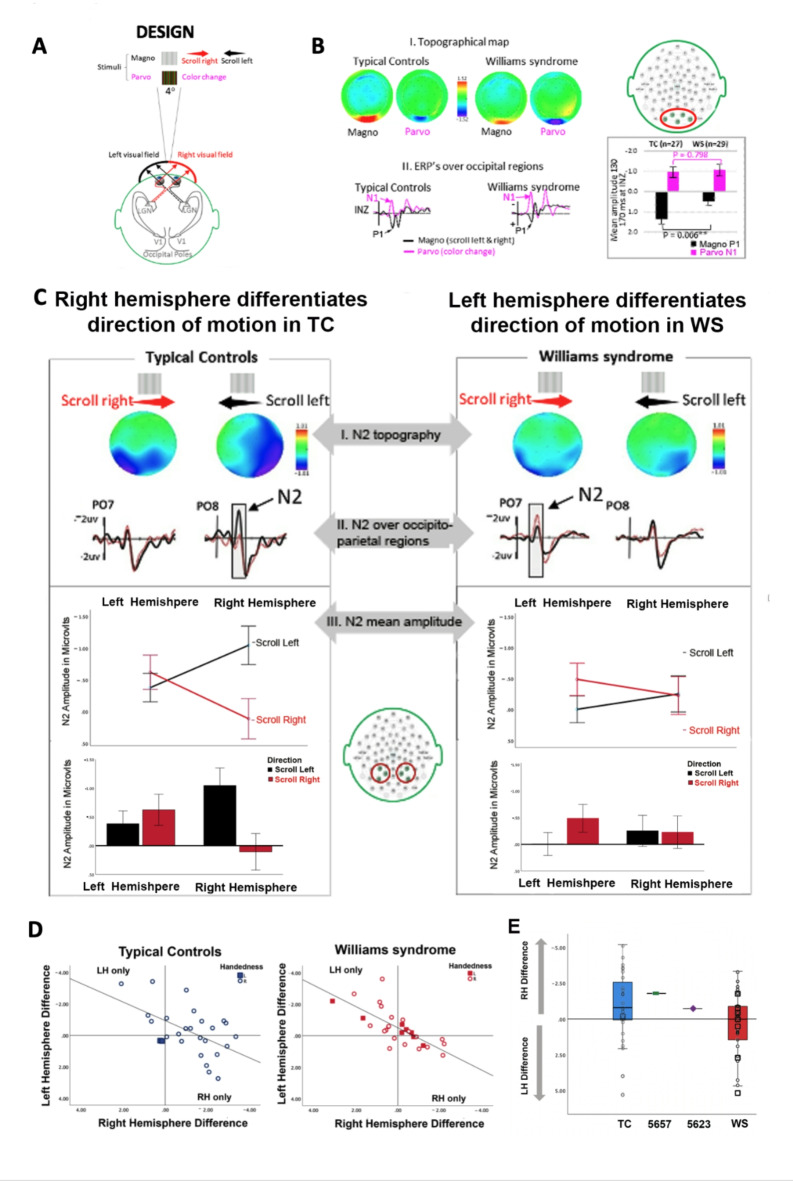
Laterality for direction of motion is reversed in WS. (**a**) Experimental design: Motion and color change stimuli (see methods) were presented to the central visual field to differentially activate brain activity to magnocellular (M) and parvocellular (P) pathways. (**b**) i. Topographical maps show differential distribution of brain activity to M and P stimuli for TC and WS. ii. ERPs and bar graphs show brain activity to movement (M pathway, black lines/bars) but not color (P pathway, pink lines/bars) is reduced in WS relative to controls. (**c**) Top Illustrates ERPs and the motion sensitive N2 to scroll left (black lines) and scroll right (red lines) over P07 and P08, located over left and right hMT. Comparison of N2 amplitudes to scroll left (black lines/bars) vs scroll right (red lines/bars) shows that for TC only the RH sites are sensitive to scroll direction, whereas for WS only the LH sites are sensitive to direction of motion, suggesting laterality is reversed in WS. (**d**) Reversal of hemispheric control in motion detection in WS. In both TC (left) and WS (right), the magnitude of the left hemisphere detection of motion was inversely proportional to detection in the right hemisphere. (**e**) Box plot z-scores of individual laterality index (LI) by handedness for TC (blue) and WS (red). Partial deletion cases (5657, 5623) are shown as well. LI indicates size of N2 difference in microvolts to contralateral vs. ipsilateral direction of motion, RH > LH (negative values), RH = LH zero, LH > RH (positive values). In all ERP figures negative voltage is plotted up and larger N2 amplitudes are indicated in negative voltage as larger.

We developed a paradigm that consistently elicits RH lateralized brain activity to direction of motion in TC and is suitable for testing individuals with WS (Supplementary Table 1, Fig. 1A, Methods, and Fig. 3). This paradigm involves presenting a spatial frequency gradient that appears to move in either a leftward or rightward direction, and by measuring the responses of the RH and LH separately and simultaneously, it provides the capability to compare the strength of RH and LH specialization in TC versus WS for direction of motion and allows us to assess the contribution of gene specific genetic expression to hemispheric lateralization.

The visuospatial (RH) deficits in WS and other neurodevelopmental disorders are thought to involve vulnerability of the dorsal stream pathway, that predominantly receives input from the magnocellular (M) pathway^35^. To evaluate the importance of the M visual pathway to the possible shared damage in neurodevelopmental disorders, it was important to confirm that the stimuli were selectively affecting the M pathway. M and P (parvocellular) are multisynaptic neuronal pathways of the visual system, originating from the retina, and projecting through the lateral geniculate nucleus (LGN) to the primary visual cortex (area V1)^35,37^. The M system responds to low spatial frequencies and movement, whereas the P system is sensitive to high spatial frequencies and color perception^38,39^. In adult controls and macaques, direction of motion is mediated in right dorsal visual area V5/ hMT (Medial Temporal) predominantly receiving input from the M pathway^40,41^. Therefore, we first ensured that we were stimulating the M pathway by using the stimuli presented to the central 2 degrees of the visual field. To do this, high density event-related potentials (ERPs) were recorded to motion (the M pathway) vs color change (the P pathway) stimuli presented within 2 degrees of central fixation (Fig. 1A, methods). M stimuli consisted of a stationary low spatial-frequency grating that appeared to scroll to the left or right for 100 ms. P stimuli consisted of high spatial frequency isoluminant red and green bars that switched colors.

**Fig. 2:**
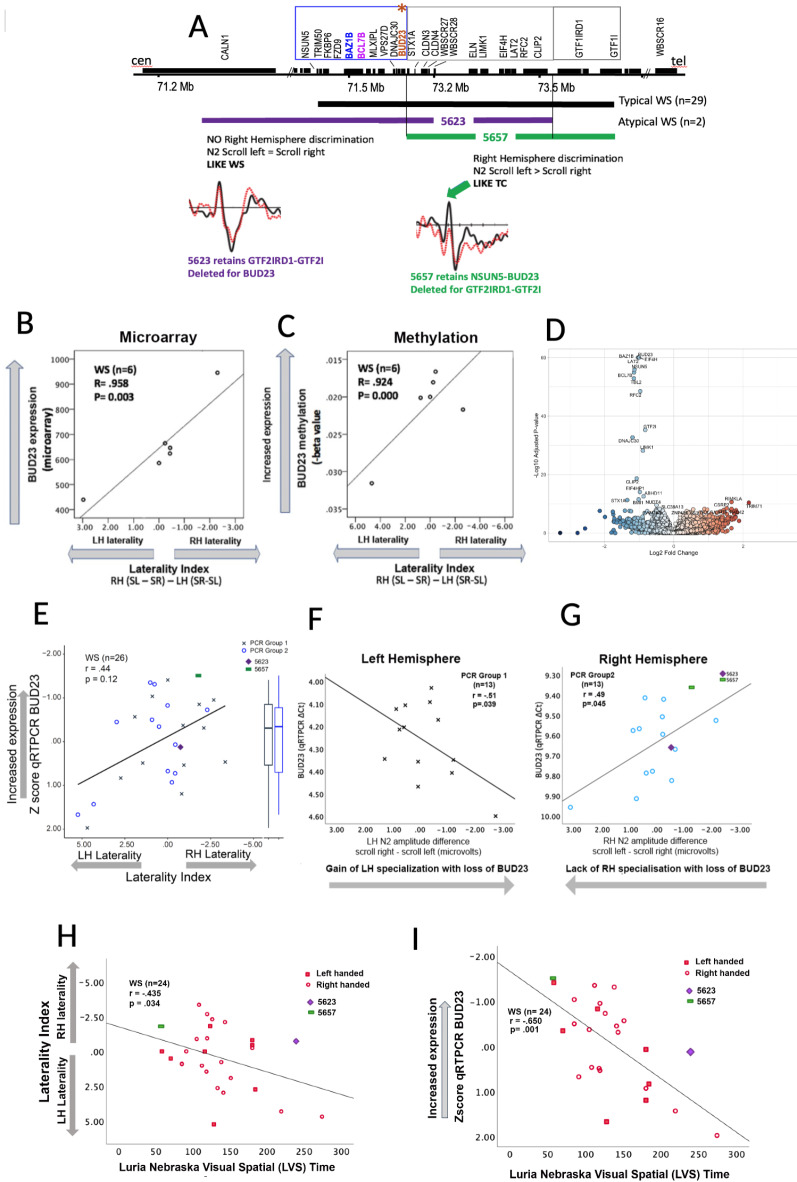
(**a**) Partial deletion cases narrow down the subgroup of genes (NSUN5-BUD23, blue box) implicated in the reversed lateralization in WS. Participant 5657 shows the right hemisphere (RH) specialization pattern consistent with controls, whereas subject 5623 shows a lack of RH specialization, but no LH specialization unlike TC or WS. (**b**, **c**) Hypothesis generation from previous data showing correlations lower expression of BUD23 is linked to loss of RH specialization and increase of LH specialization for direction of motion for microarray and methylation data. (**d**) Volcano plot of genome wide-expression analysis of mRNA isolated from WS versus TC lymphoblastoid cells. BUDS23, BCL7B, BAZ1B gene transcripts are among most significantly dysregulated transcripts. (**e**) Correlation of BUD23 expression with reversed LI in two independent replication cohorts (n = 26) by quantitative RT-PCR. (**f**) Correlation of BUD23 expression with left hemisphere specialization in PCR group 1. (**g**) Correlation of BUD23 expression with lack of right hemisphere specialization in PCR group 2. (**h**) The LI showed LH > RH specialization in WS and was correlated with increased time (poorer efficiency) in mental rotation. (**i**) Decreased expression of BUD23 was correlated with poorer efficiency in mental rotation.

For these studies we assembled a unique cohort of well-characterized individuals with the full deletion for WS recruited through a large multicenter collaborative research project (n = 29; ages 18–52, 15 female; see Supplementary Tables 1 & 2, methods). The control cohort (n = 27) was matched by age and ethnicity. In addition, we examined 2 individuals with partial deletions in the WS region. The phenotypic map of the full WS deletion as well as breakpoints for the two individuals with partial deletions is shown in Fig. 2A.

### Hypoactivation of the magnocellular pathway in WS

Consistent with previous ERP findings for TC^42,43^, we found that ERPs to apparent horizontal motion (M pathway) and red vs green color change (P pathway) stimuli differed in timing, amplitude and distribution (Fig. 1B). The main effect of condition for amplitude was F(1,54) = 65.76, *p* = 0.000; and for latency, F(1,45) = 8.429, *p* = 0.002. For both TC and WS, M stimuli elicited a P1 (black lines) and P stimuli elicited an N1 over central occipital sites, with the P1 for M (motion) peaking at 155 ms and the N1 for P (color change) peaking earlier at 148 ms. For WS relative to TC, there was selective attenuation of the P1 to M (motion) stimuli; (t(54) = 2.838, *p* = 0.006), but not the N1 to P (color change) stimuli; (t(54) = 0.257, *p* = 0.798). There were no significant group differences in latencies for M-P1 or P-N1 components (all were above *p* = 0.05).

### Laterality for direction of motion is reversed in WS

Hemispheric specialization for direction of motion was assessed by comparing ERPs to leftward vs rightward apparent motion for the M stimuli. We used an established lateralized electrophysiological marker (N2)^44,45^ sensitive to movement presented to the central visual field recorded over left and right temporo-occipito-parietal regions (Fig. 1C, middle). Consistent with traditional hemifield presentation studies^44,46^ the N2 latency peaked at 190 ms and the N2 amplitude was asymmetrical being larger over the hemisphere contralateral to the direction of motion, F(1,54) = 39.071, *p* = 0.0001, scroll direction x hemisphere interaction (Fig. 1C, top). That is, apparent movement towards the left (i.e., scroll left, SL) elicited N2 amplitudes that were larger over the right than left hemisphere sites (RH, LH); whereas motion towards the right (i.e., scroll right, SR) N2 amplitudes were larger over the LH than RH sites. Notably, the size of the N2 asymmetry was smaller for WS than TC, scroll direction, F(1,54) = 4.809, p.033; scroll direction x group interaction; F(1,54) = 8.487, *p* = 0.005 (Fig. 1C, bar graphs).

However, we were interested in the finer level of hemispheric specializations, that is, the degree to which each hemisphere is differentially sensitive to direction of motion, rather than quantifying only the relative amplitude of the RH versus LH ERP asymmetry. Consistent with the recent laterality consensus initiative^47^ we defined *laterality* as the extent to which the two hemispheres were differentially sensitive to direction of horizontal motion. Here we define *sensitivity* within each hemisphere as the magnitude of the N2 amplitude difference to changes in direction of motion that are larger in the contralateral vs ipsilateral direction. In this case, the two hemispheres can both be very “sensitive” or not at all, but if they are equally sensitive, they are not “specialized”. Thus, to assess laterality, we compared N2 amplitudes for leftward vs rightward motion separately for the left versus right hemisphere sites. For TCs only the right hemisphere showed sensitivity to motion direction (Fig. 1C, middle left side); specifically, RH sites, t(26) = − 4.470, *p* = 0.000, LH sites, t(26) = 0.836, *p* = 0.411. In contrast, WS exhibited a reversed pattern with only left hemisphere sensitivity (Fig. 1C, middle right side); left hemisphere sites, t(28) = 2.390, *p* = 0.03, RH sites, t(26) = 0.113, *p* = 0.911.

### Inverse interhemispheric sensitivity for motion direction in WS and TC

The graphs in Fig. 1C illustrate findings at the group level. To determine whether differences in the size of RH versus LH sensitivity were related, these were plotted for each individual and correlated for the group. Figure 1D illustrates the relationship of LH (Y axis) versus RH (X axis) sites in the size of their N2 response (sensitivity) to contralateral versus (minus) ipsilateral motion for each participant (TC right; WS middle component of Fig. 1D). Remarkably, (Fig. 1D) the larger the magnitude of the RH sensitivity, the smaller the LH sensitivity, showing a true inverse hemispheric strength in TC, r = − 0.537, *p* = 0.004. The inverse correlation in RH vs LH sensitivity was even more pronounced in WS, r = − 0.730, *p* = 0.0001).

### The laterality index: a metric to quantitate the relative size of the RH versus the LH specialization

The Laterality Index (LI) was first introduced as a metric to quantify asymmetry of brain volume or activity^47^. Here, we applied this to reflect the relative contribution of RH versus LH sensitivity to direction of motion. We calculated the LI by subtracting the differences in N2 amplitude to motion change for the RH (scroll leftward—rightward direction) from the LH (scroll rightward – leftward direction) sites. An LI of 0 indicates equal N2 amplitude differences over RH and LH sites, signifying a lack of hemispheric specialization (LI = 0; RH = LH). Negative values indicate RH > LH laterality, while positive scores indicate LH > RH laterality. Individual and group laterality scores are shown in Fig. 1E. In TC, 84% of right-handed individuals exhibited RH specialization, whereas in WS, 62% showed LH specialization, regardless of handedness (WS vs TC, t(54) = 2.193, *p* = 0.03). No significant correlations were found between LI and IQ, age, sex, or handedness in WS or TC, suggesting specificity of the reversed laterality for direction of motion in WS findings (Table 2).

## Results: genetics

The reversed hemispheric specialization for motion direction in WS illustrates what might be learned from genetically altered brains to inform the leap from DNA variants to single genes with quantitative outcomes that covary with brain function and behavior in *single individuals*. Therefore, we asked whether in single individuals with WS and the same gene copy number, the level of transcription of single WS deleted genes predicted their hemispheric reversal of specialization.

It has been challenging to identify genes that vary with an individual’s cognition as required for clinical applications. In contrast to GWAS studies, small cohorts with well-defined quantitated traits and consistent genetic mutations are powerful models for drilling down to single genes with large effect sizes. Therefore, we used multiple small cohorts and addressed the need for replicate studies by separating hypothesis generation from statistical testing. To do this, we narrowed a gene-specific hypothesis for the reversed hemispheric specialization using two independent small groups and two individuals with unique overlapping deletions^23,26^ (Fig. 2A).

For hypothesis generation, we used three approaches. The first two measured gene expression, first with RNA transcript levels (N = 6) and then with DNA methylation using a largely (5/6) independent cohort (N = 6), for each of the 11 WS deleted genes detected previously^48^. For each group, we then correlated the individual’s transcript or methylation level with their ERP LI for direction of motion (right lateralized in controls and left lateralized in WS as above). For this phase, these were the only cohorts available with pre-existing data for both ERPs and expression (Illumina p450 array) or methylation data.

We found that three WS genes, *BUD23*, *BCL7B*, *BAZ1B,* each had significantly lower transcript levels in WS versus TC as determined by quantitative microarray^48^ analysis (Fig. 2B; Supplementary Fig. 1). The gene expression data discussed in this publication have been deposited in NCBI’s Gene Expression Omnibus and are accessible through GEO Series accession number GSE285666 (https://www.ncbi.nlm.nih.gov/geo/query/acc.cgi?acc=GSE285666). The expression levels of these three genes (n = 6) also exhibited a significant negative correlation with decreased RH sensitivity for direction of motion in WS subjects (r = − 0.96, *p* = 0.002; r = 0.95, *p* = 0.003; r = 0.93, *p* = 0.005, respectively) as well as a positive correlation with the LI for WS (r = 0.92, *p* = 0.01; r = 0.86, *p* = 0.029; r = 0.83, *p* = 0.04), that is, decreased expression was correlated with left greater than right lateralization (Fig. 2B; Supplementary Fig. 1, Supplementary Table1).

The second method revealed that increased methylation of *BUD23* (cg 12,099,727, located 40 nt upstream of exon 1 in the putative BUD23 promoter) and decreased methylation of *BCL7B* (cg 09,265,173, also located in its putative promoter, 180 bp 5’ of exon 1), but not of *BAZ1B* were significantly related to the LI index of hemispheric reversal for direction of motion (for *BUD23* Fig. 2C in text; *BUD23* r = − 0.942, *p* = 0.005; *BCL7B* at cg09265173 r = 0.898, *p* > 0.015; *BAZ1B* at any cg site, *p* > 0.2 (Supplementary Table 2)). Increased methylation of *BUD23* and decreased methylation of *BCL7B* were also correlated with RH loss, and negatively correlated with LH gain (Supplementary Figure S2; worksheet data Supplementary Table 4). Only correlations with *BUD23* survived multiple comparisons for each of the three, forming the basis for the hypothesis that decreased transcription and/or increased methylation of *BUD23* was related to reversal of hemispheric specialization for motion direction in WS (Supplementary Table 2).

As a third independent test of *BUD23* as a candidate for hemispheric reversal of specialization to motion detection, we quantitated transcription in two rare individuals with WS and overlapping partial deletions discordant for *BUD23* (Fig. 2A)*.* These included 5623, a high functioning individual, (IQ FSIQ 88) deleted for *BUD23* and all WS genes except two (*GTF2I, GTF2IRD1*). The second individual, 5657 (IQ FSIQ 79, more WS typical) carries a smaller deletion of genes telomeric to *STX1A*, but maintains two copies of *BUD23, BCL7B, BAZ1B*, and all genes centromeric through NSUN5 (Fig. 2A). These findings were consistent with the microarray and methylation hypotheses that genes at the centromeric end (*NSUN5-WBSCR22/BUD23*, blue box) might be involved in the reversed lateralization for direction of motion in WS. ERPs to direction of motion from participant 5657 shows the right hemisphere (RH) specialization pattern consistent with controls, whereas participant 5623 shows a lack of RH specialization but no LH specialization unlike TC or WS. For 5623, qRTPCR (below) confirmed decreased *BUD23*, in the middle of the full deletion WS range (z = − 0.12, n = 26 WS), in contrast to 5657, who retains *BUD23* with TC range expression greater than all WS (z = 1.5, Fig. 2E). The ERP results indicated that 5657, not deleted for *BUD23*, was well within the normal range for multiple ERP features despite the larger number of genes deleted and his lower function. His RH > LH N2 asymmetry and RH N2 sensitivity to leftward vs rightward detection of direction of motion was typical of TC (Fig. 2A). Moreover, his LI was well within the normal and outside the WS range, consistent with his LI showing greater RH than LH specialization (Figs. 1D and 2G), coincident with his two copies of *BUD23*. In contrast 5623, deleted for *BUD23* fell within the WS distributions despite high function, showing a WS type N2 asymmetry, LH sensitivity (Figs. 1D and 2A), with no difference to leftward versus rightward motion over the RH, coincident with his BUD23 deletion. In summary, each partial deletion case showed patterns consistent with BUD23 copy number across genes, brain, and behavior, with individual 5657 at the high end for BUD23 expression, RH laterality and MR efficiency, and individual 5623 in the middle range on all three measures.

In summary, the hypothesis generating data were most consistent with the involvement of BUD23 in the reversal of WS asymmetry for detecting direction of motion in WS. That is, only BUD23 transcript level and putative promoter methylation correlated with RH loss and LH gain of specificity as well as with the LI. That is, decreased *BUD23* was correlated with increased LH specialization, and increased BUD23 with RH specialization. The data are compatible with a likely lesser contribution of BCL67B and BAZ1B to WS asymmetry.

### BUD23 expression is correlated with reversed hemispheric specialization in WS with full deletions

To statistically validate the role of *BUD23* in reversed hemispheric specialization, we utilized RNA isolated from LB cells derived from a larger independent cohort of 26 individuals with WS and quantified *BUD23* transcription levels using qRTPCR. Because *BUD23* is decreased in WS along with reversed asymmetry, we predicted that decreased expression of *BUD23* would be correlated with the loss of RH specialization and a left greater than right LI, and accordingly, used directional one-tailed tests for these correlations. To further address replicability, the cohort was divided and analyzed in two groups (n = 13, n = 13) selected by date of acquisition. The data from the two separate cohorts were combined using z scores to address possible batch effects. Figure 2E shows that the decreased transcript level of *BUD23* (measured with qRTPCR) in the combined two PCR groups (n = 26), correlated with the laterality index (r = 0.465, *p* = 0.013, two-tailed). Further, decreased *BUD23* was independently correlated with both the loss of RH specialization (r = − 0.441, *p* = 0.019, two-tailed, n = 26) and gain of LH specialization (r = 0.423, *p* = 0.012, two-tailed, n = 26) for motion direction detection, substantiating the results from the smaller hypothesis generating cohorts that implicated *BUD23*. Repeated experiments (PCR Groups 1 and 2) with multiple control transcripts (Figs. 2F,G, Supplementary Table 3) showed that decreased expression of *BUD23* was correlated with both the gain of LH specialization for PCR Group 1 (n = 13, r = − 0.507, *p* = 0.039, one-tailed) and loss of RH specialization for PCR Group 2 (n = 13, r = 0.489, *p* = 0.045, one-tailed) independently.

### Mental rotation: a general role for laterality and BUD23 in RH specialized deficits?

The Luria-Nebraska^49^ task was used to measure mental rotation, decreased in WS. Although both outcome measures, viz., efficiency and accuracy, were decreased in WS, Fig. 2H,I shows that only decreased efficiency (Fig. 2H) but not accuracy (*p* > 0.05) was correlated with the LI (n = 26, r = − 0.402, *p* = 0.034), with the magnitude of the LH sensitivity (n = 26, r = − 0.529, *p* = 0.008) and with decreased *BUD23* expression (Fig. 2I) (n = 26, r = 0.641, *p* = 0.001).

## Discussion

In summary, the results of three lines of converging evidence for hypothesis generation (quantitative transcription, DNA methylation and atypical deletions), followed by testing of quantitative expression in a larger cohort using qRTPCR, indicate the involvement of *BUD23* in reversed laterality in WS. We propose that the independent correlation of decreased expression level and promoter region methylation of *BUD23* with poorer performance in two RH specialized tasks together with correlation of each with LH gain in sensitivity, indicates RH dysfunction with reassignment of a part of the task to the non-optimized LH circuitry. Moreover, each partial deletion case showed patterns consistent with BUD23 copy number across genes, brain, and behavior, with individual 5657 at the high end for BUD23 expression, RH laterality and MR efficiency, and individual 5623 in the middle range on all three measures. Thus RH dysfunction in WS is indicated not only as loss of function but also as reversed brain laterality related to *BUD23*.

A fundamental question of laterality is how the RH and LH develop a distinct but cooperative effect on a given function that is advantageous to the organism. It is unknown whether hemisphere-specific activation related to a behavior has any relationship to co-activation or inhibition of the contralateral hemisphere. The results (Fig. 1D) suggest an inverse relationship. The magnitude of the ERP sensitivity to motion direction over the RH sites was inversely proportional to the magnitude of sensitivity over LH sites. This striking inverse strength was observed in both TC and in WS and suggests inverse coordinate regulation of lateralized hemispheric function. There is no reason why this would be the case if the two hemispheres were equipotent for processing direction of motion. This is supported by the results of a recent fMRI study reporting that hemispheric specialization for multiple lateralized functions was related within a hemisphere and reversals affected the full set of functions^50^. Moreover, specialization for each function was mirrored across the two hemispheres with poorer performance associated with atypical or reversed laterality^50^. Taken together with the current report, the results suggest a coordinate interhemispheric mechanism that mediates laterality and involves BUD23.

### Reversed lateralization

Of particular interest was that the group results suggested laterality for direction of motion is specialized to the RH in TCs and is reversed to the LH in WS (Fig. 1C). It is widely reported that individuals with neurodevelopmental disorders show disturbed but also less lateralization which has been reported as non-specific loss of specialization. In contrast, reversals in laterality are rare and support specific genetic alterations in pre- or post- natal development and plasticity of the neural systems involved. The strong inverse interhemispheric mechanism implicated above for both TC and WS helps illustrate that WS are not simply “less lateralized”. In fact, the results shown in Fig. 1D (upper right quadrant) suggest that in WS, the RH and LH may be more tethered, reflecting decreased circuit flexibility. These results show that, in contrast to TC, no individuals with WS show large amplitude differences to direction of motion over both RH and LH simultaneously. Moreover, in WS the inverse loss of RH and gain of LH sensitivity argues for a true hemispheric reversal of specialization rather than a sole loss of RH function due to developmental damage.

### Mental rotation

Mental rotation (MR) is one of the most well-studied higher order cognitive tasks that are abnormal in WS^51,52^ and also thought to involve RH specialization associated at least in part with the same brain area involved in direction of motion, viz*.*, area V5/MT, validated by fMRI studies of TC^51–55^. The Luria-Nebraska^49^ task is a gold-standard measure of mental rotation abilities in which reaction time is independent of accuracy, the former measuring mental *efficiency* versus the latter, *efficacy* of the neural circuit^54^. Moreover, the Luria-Nebraska task is particularly useful for WS as the outcomes are without floor effects that are more evident in other MR tasks. We reasoned that the RH to LH reversal of specialization found for direction of motion, might also affect other RH specialized functions, not only related to RH loss but also in part related to decreased efficiency of LH circuitry when subsuming tasks normally performed by the more efficient but in WS, impaired RH circuitry. The correlation only of increased time but not decreased accuracy on the Luria-Nebraska, with both the LI (Fig. 2H), with gain of LH sensitivity, and with decreased BUD23 (Fig. 2I). This suggested decreased efficiency for mental rotation possibly due to LH mediation of the task.

In conclusion, three observations suggest that lateralized brain activity is regulated in a similar manner in WS and TC, but is genetically modified in WS: inverse hemispheric strength for direction of motion in WS and TC, hemispheric reversal for motion direction in WS and the correlation of the reversal index, the LI, with deficits in MR efficiency, a second RH function. An important implication of these results is that, for those RH lateralized phenotypes related to hMT, viz., motion direction and mental rotation, the mechanism of deficits may involve reversed laterality.

### Dorsal stream vulnerability and the M pathway

The results inform an important if controversial hypothesis suggesting that abnormal input from the M pathway accounts for “dorsal stream vulnerability” in neurodevelopmental disorders including ASD^56,57^ Schizophrenia^58^ and Dyslexia^59,60^. The basic tenet, that hypoactivation of the M pathway subserves visuo-spatial deficits and may be responsible for the reversed lateralization observed here, was only partially supported by the results. Although we observed selective hypoactivation of the M pathway, the degree of hypoactivation was not correlated with the LI, gene expression, or MR. The lack of significant correlations with ERPs (P1) recorded over primary visual cortex could be the result of WS deleted genes affecting the fetal pathway that utilizes predominantly the inferior medial pulvinar and superior colliculus, rather than the adult pathway that utilizes the LGN and V1^61^ .

### Parcellation of genetic contributions to visual-spatial deficits in Williams syndrome

One of the major challenges in identifying the genes involved in lateralization has been the identification of reliable and precise lateralized phenotypes. Here we identified a RH specialization for direction of motion in 84% of right-handed TCs. This is equivalent to percentages observed for LH specialization for language, and surpasses even the most reliable index of RH asymmetries, the N170 ERP to faces^62^. By combining ERPs and genetics, the findings provide novel insights that parse genetic contributions to the three WS visual-spatial phenotypes associated with dorsal stream vulnerability and RH dysfunction. Specifically, direction of motion and mental rotation both involve area hMT in contrast to visual-spatial construction that does not, suggesting the former two might share genetic contributions that are distinct from the latter. The data above support this genetic distinction. The LI measured here for direction of motion almost certainly reflects activation in left and right hMT in the dorsal stream in that, despite the limited spatial resolution of ERP’s, the location of hMT on the lateral cortical surface, make it ideal for using ERPs to record its activity at the scalp. Further, the role of hMT in processing direction of motion is one of the few findings undisputed in the literature^63,64^. This source localization of direction of motion and the sensitive ERP (the N2), to area hMT, has been strongly supported by previous work using simultaneous fMRI and MEG^41^and TMS^40^. Consequently, the data presented here stratify WS with full deletions and correlate quantitative transcription of BUD23 and chromatin remodelers (BAZ1B, BCL7B, and GTF2I), as well as DNAJC30^28^ with hMT related direction of motion and mental rotation but not with visual-spatial construction, a third RH lateralized function that is not related to hMT activity (Table 2). In summary, the data from WS suggest that deficits in RH lateralized functions may be related to more than one gene or mechanism, with BUD23 transcription contributing to hMT lateralized functions and GTF2IRD1 and GTF2I to visual-spatial construction in WS, supported by studies of partial deletions with or without a given gene^24,65^. Although RH visual-spatial construction lateralized to the parietal lobe in TC, this is unknown in WS and It will be important to utilize hemisphere specific measures of visual spatial function to assess the extent to which hemispheric reversal may contribute to additional circuitry in these studies as well as in recent associations of LIMKinase1 with structural variation of the RH Internal Parietal Sulcus Gray matter^66^. The significance of the results is in discovering a role for BUD23 in brain laterality and suggesting mechanism-based candidates for neuropsychiatric and behavioral disorders associated with perturbations of laterality.

### BUD23 in biology and development

These findings are timely for elucidating neural and genetic mechanisms of brain lateralization given the recent emergence of BUD23 and its central roles in cell biology, epigenetics and brain development. BUD23 is ubiquitously expressed in brain from early embryogenesis and acts primarily in ribosomal biosynthesis, a process that is highly conserved from yeast (Saccharomyces cerevissiae) through humans and is responsible for synthesis of all cytoplasmic proteins that together consume 60% of a cell’s energetic resources. BUD23 is one of hundreds of accessory proteins and small RNAs that are involved in processing ribosome precursors prior to transport to the cytoplasm but are not part of the mature ribosome. BUD23, together with its co-activator TRMT112, acts both as a guanosine methyltransferase of the 18S rRNA (G1639)^67^ and in the non-catalytic maturation of the pre40S particle precursor of the small ribosomal subunit (SSU). Although its major function is in generating cytoplasmic 40S ribosome subunits, decreased BUD23 has also been associated with decreased mitochondrial energy production in cardiac myocytes, thought to be related to its effect on decreasing cytoplasmic synthesis of nuclear encoded proteins required for mitochondrial ribosomes^68^ . Consequently, the striking evolutionary conservation of BUD23 and its dosed effects on ribosome biosynthesis and mitochondrial dysfunction support a phenotypic role in humans.

Although speculative, a possible relationship of BUD23 to brain asymmetry may be through the function of cilia, mutations of which are a major cause of human disorders of somatic asymmetry^69^ although their role in CNS asymmetries is unclear^70^. Recent work showing that ciliary function in neural cells can be compromised by mitochondrial dysfunction^71,72^ and mutation^73^ is of interest. Therefore, taken together with its effects on mitochondrial dysfunction^68^, the BUD23 decrease in WS may also fail to meet the high energy demands required for normal ciliary function during development, leading to a disruption of right-left axis laterality seen here and possible consequent effects on cognitive or behavioral function^74^. Although regulation of ribosome biosynthesis may be a primary function by which BUD23 affects asymmetry, recent work has suggested additional roles of BUD23 in that may also contribute to diverse WS phenotypes including regulating glucocorticoid receptor function and target gene histone methylation^75^, and in facilitating viral infectivity by tethering viral proteins to chromatin^76^. Curiously, the highly conserved sequence and function of BUD23 may provide a prototype for human dosage sensitive genes in that regardless of phenotype, the effects of decreased BUD23 are dosage sensitive in model systems from yeast through humans^68,75,77^.

### DNA methylation and asymmetry in WS: Role of BUD23 Dosage

Two independent lines of evidence now link BUD23 methylation to altered Right-Left asymmetry in WS, the first from the hypothesis generation data (Fig. 2D) showing that methylation of the CpG located in the BUD23 upstream promoter region (40nt upstream of the start site) was correlated with the reversal LI. This is supported by independent previous work showing increased genome wide methylation in WS^28,78^. The subsequent WGCNA (Weighted gene co-methylation network analysis) of WS versus control blood samples^78^identified 14 co-expression modules of which Module #8 contained the only WS gene, BUD23 (Fig. 2H) for which Enrichr^75^ Kuleshov 2016 analysis (version 2017b) revealed functional association with Right-Left asymmetry (Fig. 2H). This supported our correlating the BUD23 putative promoter CpG methylation with the LI (hemispheric reversal) in WS. Further, we note recent work^79^ suggesting that BUD23 may be directly involved in WS genome-wide methylation by virtue of its role as a dosage related inhibitor of Dnmt3a, the enzyme responsible for human de novo DNA methylation of developmental genes^80^.

The decreased brain BUD23 in WS^48^ could therefore account not only for decreased ribogenesis and consequent decreased cytoplasmic and mitochondrial protein synthesis^68^, but also for increased genome wide methylation^78^ through its predicted decreased inhibition of Dnmt3a^79^. These multifunctional dosage related roles in transcription and methylation could contribute not only to the LI correlation with both BUD23 transcript level and methylated promoter CpG reported here, but also to the association of the WS co-methylation module^78^ with R-L axis asymmetry. Although further WS deleted genes likely contribute to methylation and chromatin remodeling^28^, the correlation with the LI is the first report of a functional association in WS. We note that there are two distinct forms of epigenetic regulation related to BUD23, DNA methylation (CpG) in the promoter region influencing transcription, and methylation of the 18S rRNA involved in ribosome function by its protein product. Taken together, the results suggest a possible role for epigenetic regulation involving BUD23 in WS asymmetry and provide a possible mechanism for the post-natal left–right axis shifts in direction of motion seen in deaf and hearing native signers^81^ as well as in other WS cognitive deficits^82^.

### Hypothesis: An evolutionarily conserved role for BUD23 in asymmetry for direction of motion?

Given the inaccessibility of human brain samples, it is not surprising that little is known of the genetic circuitry mediating human laterality^16–18^. However, our further analysis of genetic datasets^83,84^ that integrate model organisms across evolution, suggested a possible evolutionary link. We determined that the association of methylation module 8 with R-L CNS axis asymmetry was based on GO (Gene Ontology) term 0,035,545 that is derived from work elucidating the genetics of asymmetry in the nematode, *Caenorhabditis elegans* (CE)^15,85–87^, a major model of CNS right-left asymmetry. It is of interest that BUD23 is highly homologous in CE and humans and its major functions are highly conserved including its role in SSU ribogenesis^88,89^, in de novo methylation^79,80^, in intergenerational hormesis^90^ and possibly in asymmetry. Specifically, in CE asymmetry of the right (ASER) and left (ASEL) taste neurons, the initiating event involves Glp1-Notch signalling^85^. It is of interest that BUD23, regulates the glucocorticoid receptor (GR)^75^ that, although in different biological contexts, in turn regulates Glp1 (Glucagon like peptide 1)^91^. Both processes are dosage sensitive^75,91^as are the mechanisms of WS. The point of interest is that cell autonomous dosage of Glp1 is a critical determinant of R-L asymmetry in CE^15,85^ in that rightness of the ASER neuron is related to high Glp1 and leftness of the ASEL neuron to low Glp1. Therefore, although speculative, previous data suggest the possibility that the decreased BUD23 observed in WS^48^ and modeled in CE, could decrease GR binding to its targets including Glp1, thereby increasing “rightness”, and decreasing “leftness”^85^ altering downstream chemotaxis and direction of motor responses to gradients^92^. Given the close to identical roles of BUD23 in three cellular systems of human and CE, it may be acting at multiple points to influence asymmetry, viz., genome wide methylation, ribosome biosynthesis, and GR regulation. It is possible that phylogenetically more recently evolved lateralities such as LH for language, may share conserved cellular elements with ancient literalities such as direction of motion but they may be more difficult to detect. Although speculative, the role of these mechanisms in asymmetry is testable in available CE models.

It will also be of interest to examine RL asymmetries in CE knockouts and hypomorphs for BAZ1B and BCL7B, the additional deleted gene transcripts correlated with the WS LI. Finally, it is intriguing that in both CE and WS, the lateralized systems both involve sensory cues important for ultimately determining movement toward or away from stimulus gradients and for survival. The steps involving inverse gene expression and strength of responses to stimuli seen in R versus L asymmetric CE neurons^15^ may be related to similar R versus L inverse correlation in laterality seen in WS and TC. Regardless of mechanism, the role of BUD23 in WS asymmetry supports examining the role of BUD23 in WS brain and on the association DNA variants of CE genes involved in asymmetry and ribosome synthesis in databases with human neuropsychiatric phenotypes.

## Conclusions

In conclusion, this work addresses the dearth of studies relating quantitative gene expression to human brain function or circuitry and begins to fill a gap in understanding the role of BUD23, ribosome variation and DNA methylation in lateralization. Such studies harnessing the power of phylogenetic information on highly conserved systems may help to elucidate poorly understood disorders of intellectual disabilities, ASD^11^ and schizophrenia^12^. The results illustrate the feasibility of parsing large effect genes for complex human behaviors at the level of the individual using quantitative behavioral and genetic measures in human genetic models. Integrating the details of cellular functions from model organisms through humans, may facilitate the leap to translational solutions, including individual outcomes required for clinical trials. These data reveal a novel quantitatively inverse hemispheric specialization for motion direction, provide a novel mechanism for cognitive and behavioral deficits, viz., perturbation of brain lateralization, and identify BUD23 as a first human transcript correlated with hemispheric specialization. In a field with few mechanisms, the results underscore how genetically altered brains may suggest new directions in human translational neuroscience. The emerging role of ribosome biosynthesis and epigenetic modification in neurodevelopmental disorders may point to a role for highly conserved cellular mechanisms in distant evolutionary models such as *C elegans* to illuminate the landscape of next steps. However rarely this happens, one must not miss it.

## Methods

### Participants

ERP study participants included 29 adults with WS with full WS deletions and 27 age and ethnicity matched typically developing controls (Table 1). Individuals with WS were recruited through a large multicenter collaborative research project. Typically developing controls were recruited through advertisements in local newspapers and fliers posted in local areas and colleges. The research study was approved by the Institutional Review Boards at Cedars-Sinai Medical Center, The Salk Institute for Biological Studies and The University of Utah. All studies were conducted in accordance with relevant guidelines and regulations. Written informed consent was obtained for all participants and/or their legal guardian(s). In each case the diagnosis of WS was determined by medical history, clinical and laboratory evidence and is then confirmed by multicolor FISH^21^. All WS participants completed cognitive measurements including IQ and visuospatial and mental rotation tests (Table 2) within 2 years of the ERP testing date and when they donated cells for LCLs generation. Participants were paid to compensate for their time and travel costs. There were no significant differences between WS and TC groups in age, t(54) = − 0.147, *p* = 0.883, or numbers of males and females, χ^2^_(1,56)_ = 0.245, *p* = 0.621. There were more left-handed WS (8) than TD (2), χ^2^_(1,56)_ = 3.88, *p* = 0.05. All participants had normal or corrected to normal vision. Vision screening was conducted using the Snellen eye chart for visual acuity as well as a subset of Ishihara color vision plates to ensure the participants could see and distinguish the stimuli. Three of the WS participants in the ERP study did not have lymphoblast cell lines. Therefore data for the qRTPCR was n = 26.

**Table 1:** Participants.

Group	N	Age	# Females	# Left handed
Typical Controls	27	18–50 (m = 32)	15	2
Williams Syndrome	29	18–52 (m = 32)	18	8
Partial deletion cases	2	36–42	0	0

**Table 2:** Cognitive variables and correlations with laterality index (LI).

	WW_VIQ	WW_PIQ	WW_FSIQ	LVS RS	LVS time	VMI
TC*	103	103	103	7.29	45	90
(sd)	10.5	11.5	10.3	1.06	32.03	10.8
WS	70.48	63.93	65.34	2.46	134	49
(sd)	(8.8)	(6.9)	(7.4)	(1.7)	47.3	(6.5)
r with LI	− 0.010	0.085	− 0.013	− 0.013	0.426	0.018
	*p* = 0.960	*p* = 0.661	*p* = 0.945	*p* = 0.945	*p* = 0.03*	*p* = 0.927
r BUD23	r = 0.292	r = 0.085	r = 0.279	r = − 0.084	r = 0.650*	r = 0.17
	*p* = 0.147	*p* = 0.680	*p* = 0.168	*p* = 0.695	*p* < 001	*p* = 0.438
5623	90	87	88	2	239	75
5657	84	78	79	4	57	54

*TC from independent sample n = 31 (due to lack of cognitive testing for TC in current study) to provide normative comparison with typical WS performance. Cognitive tests included WW = WAIS Verbal IQ (WW_VIQ), Performance IQ (WW_PIQ), Full scale IQ (WW_FSIQ), Luria visual spatial raw score (LVS RS), Luria visual spatial time (LVS time), Beery Visual Motor Integration (VMI), r with LI = correlation with Laterality Index.

To investigate individual genes involved in atypical lateralization for direction of motion, we tested two participants with overlapping partial deletions sparing different subsets of genes located at opposite ends of the typical WS deletion. Details of genetic analyses for partial deletion cases 5623 (M, right handed, 42 years) and 5657 (M, right handed, 36 years) are published elsewhere^23,26^ (see also Fig. 2A). Genetic analysis for participant 5623, confirmed deleted genes FKBP6 through CLIP2 consistent with WS, but unlike typical WS was not deleted for GTF2IRD and GTF2I. 5623 had a higher IQ and visuo-spatial spatial VMI scores than is typical of WS, but showed mental rotation performance typical of WS. Genetics for 5657 indicated deletion from STX1A through GTF2I, consistent with typical WS, but with preservation of NUNS5 through BUD23. His IQ and visuo-spatial VMI scores were typical of WS, with mental rotation accuracy better than typical WS and time to completion in the normal range.

### Cognitive testing

Standardized cognitive testing for WS participants (Table 2) included the Wechsler scale of Intelligence (WAIS-R, WAIS III depending on date of ERP testing) yielding verbal (VIQ), nonverbal (Performance, PIQ) and a general index of ability level (FSIQ). Spatial abilities were assessed using the The Luria Visuo Spatial (LVS) test for mental rotation^49^, and the Beery Visual-Motor Integration^93^. The LVS consists of a series of images depicting objects or shapes in different orientations. The LVS task is to determine if two objects are the same (one being a rotated version of the other) or different. Faster and more accurate responses across 8 items indicated better mental rotation. The Beery VMI subtest involves copying a series of increasingly complex geometric shapes to assesses the ability to coordinate visual input with motor output. The test was scored based on the accuracy and completeness of the drawings. TC participants in the ERP experiment did not undergo the same cognitive tests. As standardized norms are not readily available for the two spatial tests, comparison means were calculated from TCs from age and gender matched TCs from the larger WS multicenter program project.

## Procedure

### Event related potentials (ERP)

#### Stimuli

**Fig. 3:**
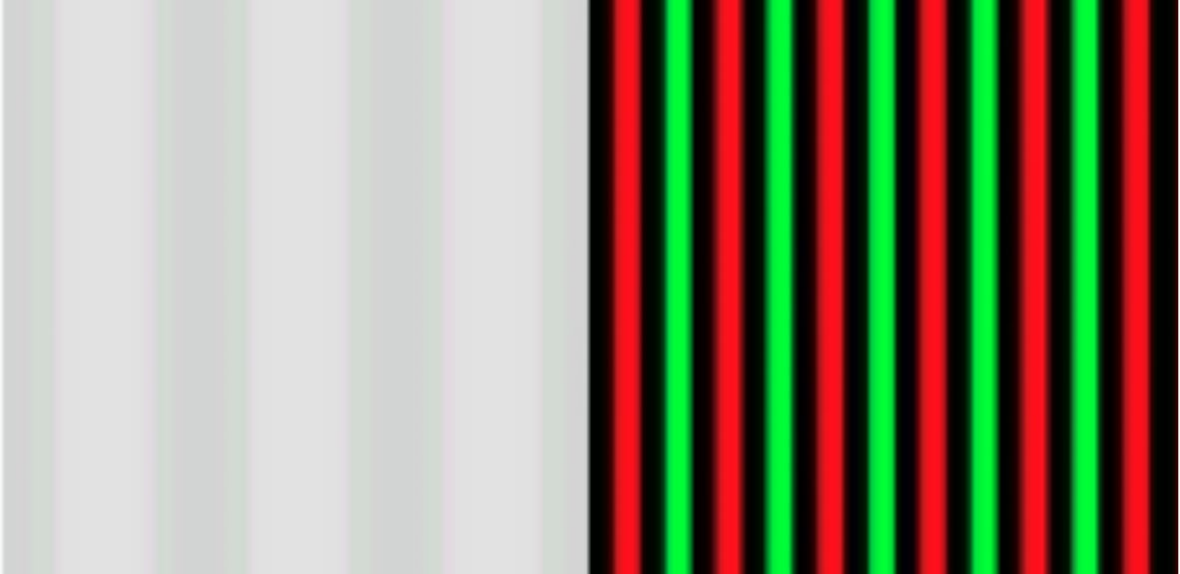
Enlarged image of stimuli used as spatial frequency gradient to detect motion (M, left image) and colour change (P, right image). The stimuli were generated using Presentation® software (Neurobehavioral Systems, Inc., Berkeley, CA, www.neurobs.com). Images shown here are from screenshots and have been enlarged for illustrative purposes. They have not been published elsewhere.

Magnocellular (M) stimuli consisted of a stationary grayscale low spatial frequency grating that appeared to scroll to the left or right for 100 ms. Parvocelluar (P) stimuli consisted of high spatial frequency isoluminant red and green bars separated by black bars that switched colors (Fig. 1A, for a larger image see Fig. 3). All stimuli were presented within + / − 2 degrees of central fixation to minimize horizontal eye movement. The stimuli (Fig. 3) were presented as a single 4.5 mm × 4.5 mm square comprised of sine wave gratings continuously visible in the central visual fields against a gray background (2 degrees each side of central fixation). The M stimuli (left) consisted of low spatial frequency gratings (0.5 cycles per degree) of light and dark gray bars with a low luminance contrast (4%). For the eliciting stimuli, bars appeared to move transversely to the right or left (random) to prevent adaptation effects (velocity 13.7 d/sec) for 100 ms. The P stimuli (middle) were isoluminant (based on the average isoluminant point for 20 adults) red and green high spatial frequency gratings (2 cycles per degree). For the eliciting P stimuli, the bars changed color (red bars changed to green) for 100 ms. To saturate the responses of the M pathway neurons to detect color change, black bars were inserted between the red and green bars^94^. Target stimuli (right) were cartoon figures that were tangential to the experiment.

The stimuli were presented to the center of a 22″ high resolution CRT computer monitor. Participants sat 36″ directly in front of the center of the monitor. The M and P stimuli were presented in two separate alternating blocks of 150 trials per block (300 trials per condition). Order of condition was counterbalanced across participants. For both the M and P stimuli the inter-stimulus interval (ISI) between eliciting stimuli was jittered with a mean duration of approximately 600 ms (+ / − 150 ms). Eye fixation was monitored by a video camera. Data acquisition was stopped if participants looked away from the center of the screen. On approximately 10% of the trials the gradients change to a color photo of a cartoon figure (target stimulus) presented for 5000 ms to provide a rest period and time to blink. The participants were instructed to press a button when they detected a target. The target stimuli were irrelevant to the experimental manipulations but insured that the participants were paying attention throughout the task. The time to complete the task was approximately 10 min. The experiment was performed using Presentation® software (Neurobehavioral Systems, Inc., Berkeley, CA, www.neurobs.com).

#### EEG recording

Electrophysiological data were recorded from a 64-channel Ag/AgCl electrode HydroCel Geodesic Sensor Net (Geodesic EEG System 300, Electrical Geodesic Inc, Eugene, OR) with an online-reference at the vertex (Cz). The EEG was sampled at 250 Hz, with a bandpass of 0.1–100 Hz. The electrooculogram was recorded from over and under the left eye to detect blinks and vertical eye movements, and from the right outer canthus to detect horizontal eye movements. Offline data were re-referenced to average activity of the left and right mastoids. Averaged ERPs were digitally low-pass filtered at 30 Hz. Event-related potentials (ERP) for M and P stimuli were segmented over an epoch of 1100 ms (starting 100 ms prior to the presentation of the stimulus), using a 100 ms pre-stimulus baseline. Separate ERP average waveforms were computed for P, M, and M scroll left, and M scroll right, for the TD and WS participants. The ERPs for each trial were checked for artifact and trials containing vertical or horizontal eye-movements or deflections exceeding ± 200 mV were removed. Across both groups an average of 60% of trials (i.e. mean of 180, range 120–300 trials per condition) were artifact-free and retained for analysis. ERPs were also computed using the artifact correction algorithm in the Netstation software and compared with averages using artifact rejection. There were no statistically significant differences and ERPs over posterior regions were identical. To rule out the possibility horizontal eye movements (HEM) could affect the relation between ERPs over RH and LH sites: HEM were minimized with brief 100 ms stimuli presented to the CVF and trials with HEM were removed from analysis using artifact rejection. Moreover, the timing, distribution, and polarity of the effect of horizontal eye movements on ERPs are inconsistent with this explanation. An additional 3 participants with WS and 3 TCs were recruited but their data excluded due to excessive artifact. A genetics lab member not connected with this study and blind to group and experimental conditions was trained to identify artifact verified the data to be removed from the analyses.

#### ERP measurements

Measurement windows were determined by starting with windows from previous literature using similar stimuli presented to the CVF with TC adults over electrode sites over the same regions^43^. The windows were adjusted to accommodate participants with WS according to best practice standards for ERP research with clinical populations^95^. First, ERP windows were determined by measuring a single grand average across all participants, centering mean amplitude windows around peaks, and by visually inspecting each average to ensure both the specific ERP peak for each participant but not subsequent components were included in the time window.

Magno vs Parvo: The M and P stimuli elicited a P1 and N1 respectively within the same time window Mean amplitude was measured from 130 to 170 ms post stimulus onset averaged across 5 occipital electrode sites (Fig. 1B, O1, I1, IZ, I2, O2).

Direction of motion (M Scroll left vs Scroll right): Apparent motion in the leftward and rightward direction elicited a negative component, N2, peaking at 190 ms over left and right parieto-occipital regions (Fig. 1C). Mean amplitude was measured from 155 to 195 ms pos-stimulus onset averaged over six electrode sites (P5, P03, PO7, P4, PO4, PO8). The N2 was followed by a large positivity, therefore the later window was determined to measure the rise and peak of the N2 but not subsequent P2.

##### Analysis

Repeated measured ANOVAs were conducted for mean amplitudes of the P1/N1 with 2 × 2 Group (TC/WS) x Condition (magno,/parvo) and for the N2 2 × 2 × 2 Group (TC/WS) x scroll direction (Leftward/Rightward) x hemisphere (Left/Right) as factors. ERP component amplitudes and latencies for movement (M) versus red/green color change (P) were analyzed in a 2 × 2 (Group x Condition) repeated measures ANOVA averaged across 5 occipital electrode sites from 130 to 170 ms post-stimulus onset.

To assess laterality to direction of motion, the N2 mean amplitudes were analyzed in a 2 × 2 × 2 Group (TC, WS) x Direction (Leftward/Rightward scroll direction) x hemisphere (Left/Right hemisphere sites).

To reduce chances of Type 1 errors, we averaged across groups of electrodes (see above) to obtain the left and right hemisphere measures.

##### Laterality index

The LI was calculated by subtracting the differences in N2 amplitudes to motion change from the contralateral minus the ipsilateral direction over each hemisphere. The formula is: N2 amplitude difference for the RH (scroll leftward – scroll rightward) minus N2 amplitude difference for the LH (scroll rightward-scroll leftward). This is shown as RH (SL-SR)—LH (SR-SL) in Fig. 2b,c,e, f, and defined in the text in the results sub-section: “Laterality Index: A metric to quantitate the relative size of the RH versus LH specialization”. We note that it may not be clear that the LI is derived entirely from the RH and LH ERP measurements acquired for each individual and that it takes into consideration both the loss or gain of specialization for each hemisphere. So, for WS, there is a loss of specialization (the difference between ERP magnitude in the RH in response to apparent movement away from versus toward the RH). This loss of specialization of the RH is accompanied by a gain in specialization in the LH, in that it responds more than in controls to movement away from versus towards it (the LH). The LI is a measure of the extent to which this specialization measured by ERP amplitude, is lost in one hemisphere and gained in the other. There is a biological variation in the gain and/or loss of hemispheric specialization as expected.

### Genetics

#### Lymphoblast cell line culture and RNA isolation

Immortalized LCLs from each of the 26 WS were generated and cultivated under standardized conditions. The cells were grown in RMPI 1640 with 10% FBS, 5% pen/strep, 5% L-glutamine, and 0.5% gentamycin. The total RNA was isolated by using the QIAGEN RNeasy Kit.

Quantitated RNA isolated from lymphoblastoid (LB) cell lines was used to compare to ERP and cognitive data because the cell growth conditions from a single cell source, could be standardized across all individuals’ lines to limit variation due to varying environment. The RNA amounts from WS are all normalized with RNA from controls grown using the same cell type, LB cell lines. WS deleted transcripts are the only transcripts consistently expressed at ~ 50% vs controls as shown in Dai et al., 2021, and it is the small variation about this mean among subjects, that is being compared with each individual’s phenotype.

#### Transcriptome analysis using Affymetrix Human Exon 1.0 ST Array

In a previous study, RNAs from lymphoblastoid cell lines derived from 34 WS and 18 TC were used to run the Affymetrix Human Exon 1.0 ST Array. The detailed method is described in our previous paper^48^. Biotinylated target was then prepared following the protocol described in Affymetrix GeneChip® Whole Transcript (WT) Sense Target Labeling and Control Reagents (Affymetrix P/N 900,652). Hybridization is performed at 45 °C for 16 h using 5 µg of biotinylated target with each GeneChip® Human Exon 1.0 ST array (HuEx-1_0-st-v2, Affymetrix). Following hybridization, non-specifically bound material is removed by washing and detection of specifically bound target is performed using the GeneChip Hybridization, Wash and Stain kit, and the GeneChip Fluidics Station 450 (Affymetrix). The arrays are scanned using the GeneChip® Scanner 3000 7G (Affymetrix) and raw data is extracted from the scanned images and analyzed with the Expression Console software package (Affymetrix). Expression Console is used to compute separate gene-level and exon-level signal estimates for the Exon 1.0 ST Array data. Exon-level estimates are derived using the PLIER (pm-gcbg) method after quantile sketch normalization. Probes sets (exons) are filtered for a ‘detected above background’ (DABG) p value less than 0.05, resulting in 313,707 filtered exons that are considered in this study. Exon-level probe sets annotations are derived from the Affymetrix NetAffx file HuEx-1_0-st-v2 Probeset Annotations, CSV Format, Release 31. Gene-level estimates are derived using the IterPLIER (pm-gcbg) method after quantile sketch normalization. Only genes with core annotation (22,012 genes) are considered in this study. Transcript annotations are derived from the Affymetrix NetAffx file HuEx-1_0-st-v2 Transcript Cluster Annotations, CSV, Release 31.

### DNA methylation analysis using Illumina Human Methylation 450 K Array

Genomic DNA isolated from whole blood samples of 39 WS and 38 TC are used to with the Illumina Human Methylation 450 K Array as directed. The detailed method for DNA isolation is described in our previous paper^48^. Genomic DNAs (500 ng, quantified using Qubit dsDNA Broad Range Assay Kit from Life Technologies) from all subjects were bisulfite converted using the Zymo EZ DNA Methylation kit (Zymo Research, CA, USA) following the manufacturers’ standard protocol. 4ul out of 10ul of eluted bisulfite converted DNA was used for genome-wide DNA methylation using the Illumina Infinium HumanMethylation450 BeadChip Kit (Illumina, CA, USA), following the Illumina Infinium HD Methylation protocol. Each bisulfite converted DNA sample was amplified, enzymatic fragmented, and applied to BeadChips. The arrays were scanned using the Illumina iScan System. The Illumina GenomeStudio Methylation Module was used to analyze methylation data from scanned array images and check the data quality. All data sets were determined to be high quality with detected CpG > 485,000 (*P*-value < 0.01). Probes with *P*-values > 0.05 were removed across all individuals. The methylation levels (β-value, i.e., the ratio of intensities between methylated and unmethylated alleles of a given cytosine) were exported to Partek Genomics Suite 6.6 (Partek, MO, USA) for computations and statistical analyses. Methylation is measured in Beta values ranging from 0 to 1 representing the percentage of methylation.

### qRTPCR of targeted genes

TaqMan Gene Expression Assays, designed for human gene transcripts (Applied Biosystems, Foster City, CA), are used for quantifying gene expression in this report. The assay mixture consists of a TaqMan® MGB probe (labeled with FAMTM dye) and unlabeled PCR primers for a specific human gene. All the Taqman expression primer sets are commercially available from ABI, and match the Affymetrix chip at exon level. First, total RNAs are reverse transcribed into single strand cDNAs using the SuperScript III Frist-Strand Synthesis system for RT-PCR (Life Technologies, USA) and used as templates for qRT-PCR of 3 WS genes (BAZ1B, BCL7B and WBSCR22), and housekeeping genes (HPRT1 and ACTB) are used as endogenous controls throughout all the PCR batches. The qRT-PCRs are performed on Applied Biosystems 7900HT system (USA) and the data are analyzed using SDS 2.3.

## Supplementary Information

Supplementary Information.

Supplementary Information.

## Data Availability

The gene expression data discussed in this publication have been deposited in NCBI’s Gene Expression Omnibus and are accessible through GEO Series accession number GSE285666 (https://www.ncbi.nlm.nih.gov/geo/query/acc.cgi?acc=GSE285666). Data are provided within the manuscript or supplementary information files for ERPs, qRTPCR and methylation (Supplementary Table 4). Cognitive data are available from d.l.mills@bangor.ac.uk upon reasonable request.
