# Supplementary material for: Brain lateralization for perceiving direction of motion is reversed in Williams syndrome and related to BUD23
Source: Sci Rep. 2025 Jun 5;15:19772. doi: 10.1038/s41598-025-94742-3 (PMC12141689; doi:10.1038/s41598-025-94742-3)
Supplement: Supplementary file 1 — Supplementary Information. [file 41598_2025_94742_MOESM1_ESM.pdf]

Supplementary Figure S1

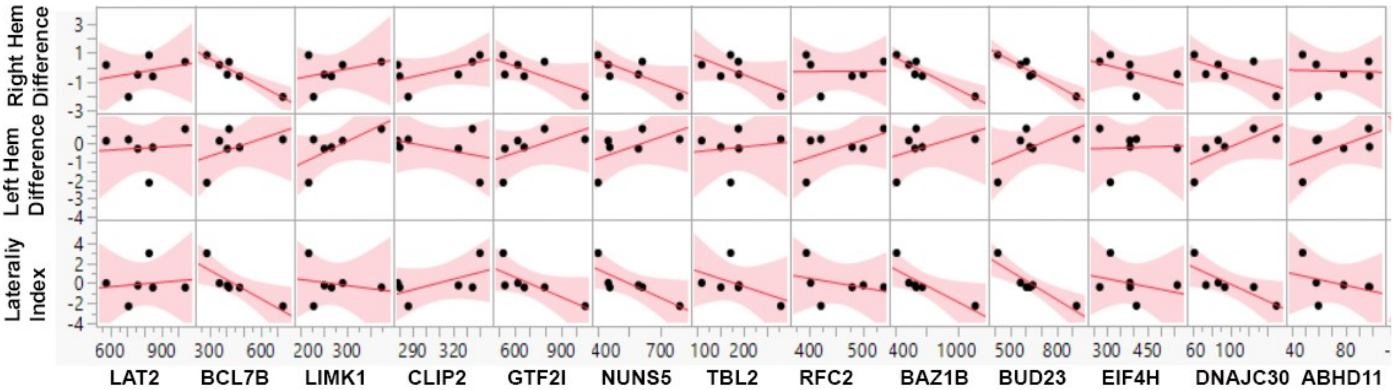

Supplementary Figure S1. Among the transcription level of 13 WS deleted genes measured by microarray in individual with WS (Dai et al., 2021), only three genes (BAZ1B, BCL7B, BUD23) show significant correlation with lack of direction of motion detection over the RH when corrected for multiple corrections p=.005.

Supplementary Figure S2. DNA methylation sites of WBSCR22/ BUD23 (Hg38)

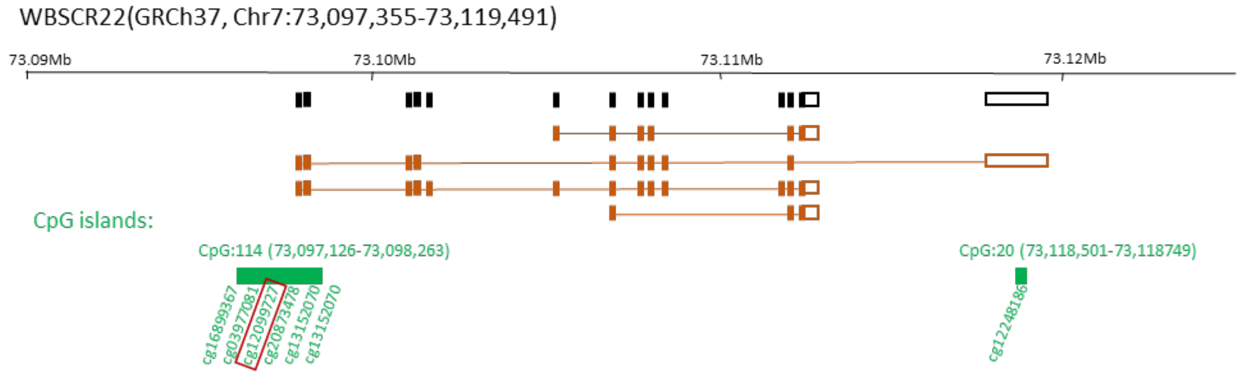

Supplementary Figure S2. Genomic locations (Hg38) of CpG methylation sites of WBSCR22/ BUD23 used for analysis vs LI.
